# Hospital and Institutional News

**Published:** 1914-03-28

**Authors:** 


### HOSPITAL AND INSTITUTIONAL NEWS.

### THE DOCTOR'S WIFE.

The article which we published last week by a doctor's wife, suggesting the formation of the Doctors' Wives Association for the purpose of supplying a free service and purchasing agency on co-operative lines, has excited the greatest interest. We have received numerous letters and signatures to the coupon which was published in the *Doctor's Wife* section of last week's issue of The Hospital, and arrangements have been made for this supplement to be reprinted. It may be obtained on application to "Co-operation," c/o The Scientific Press, Ltd., 28 Southampton Street, Strand, W.C., and all interested should write at once for a copy, as it contains valuable information as to the objects and scope of the work of the proposed Doctors' Wives Association, together with notes of the different ways in which the Association should be able to help the doctor's wife. Thus the friends of those who have already signed, and others who may learn of the organisation for the first time this week, have an opportunity of considering the proposal and forming their own judgment upon it.

### THE FIRST THOUSAND.

To those who have not seen the details of the proposal, we can only repeat the following sentence: "We find that, providing doctors' wives are prepared to co-operate in sufficient numbers and to join the Association referred to, it should be practicable to organise it in such a way as to increase the earning power of their available income now expended upon home or domestic matters by an average of from 10 to 20 per cent. Food in any form the Association cannot deal with." Co-operation in large numbers is the essence of success; a time limit is also imposed, as the Association can be formed successfully only if at least 1,000 doctors' wives join by April 18 next. These will constitute the original members, to whom special privileges should accrue. Every doctor's wife who is interested should therefore not only join herself but do her best to ensure success by making the proposal known to all the doctors' wives of her acquaintance. The eagerness with which the proposal has been welcomed in some quarters may be judged by the fact that one of the suggestions which we have already received in response to our invitation is the striking request that women doctors may be eligible for membership. Since women, when becoming doctors, do not cease, as a rule, to experience the cares of household management, there is no reason why they also should not reap the advantages of the co-operative principle by joining as members. A coupon similar to that which appeared last week will be found on page 712 of this issue. The Doctors' Wives Association, we repeat, must have at least 1,000 members by April 18 if it is to be launched successfully.

### NEW CHAIRMAN OF CHARING CROSS HOSPITAL.

Mr. George Verity has been appointed chairman of Charing Cross Hospital in succession to Mr. Robert G. V. Duff, who has resigned. The new chairman has been actively associated with the hospital for several years, and came to the work as a business man able and willing to undertake a meed of personal service by placing his energies at the hospital's disposal. He has been vice-chairman of the general purposes committee, chairman of the finance committee, and vice-chairman of the hospital. During the past year or two, and especially of late, the hospital's financial position has been improving, and hopes are entertained of re-opening before very long the closed wards. It is also felt that the removal of Westminster Hospital will add to the work which already falls upon Charing Cross, and therefore the outlook is encouraging in the best sense that opportunities are widening, and the financial difficulties of the past gradually being surmounted. Mr. Verity has always taken an interest in the Charing Cross Hospital, and the well-known firm of electrical engineers, of which he is chairman, must be among the oldest tenants on the Covent Garden Estate, their London offices being within a two minutes' walk of the hospital.

### GREAT GIFT TO CARDIFF MEDICAL SCHOOL.

The voluntary system at Cardiff is reaping the reward of the energy and skill of its main protagonists, the latest and most remarkable proof of which is the magnificent offer of an anonymous donor to complete the building and equipment of the Welsh Medical School. Lord Merthyr, as president of the University College of South Wales and Monmouthshire, has been officially informed of the details and conditions of the offer by Colonel E. M. Bruce-Vaughan, and the Council has gratefully accepted it. The gift, in the latter's words, is to provide "the necessary funds for the erection of an up-to-date public health laboratory for the city and county, and which building shall include provision for the subjects of bacteriology (and pathology if necessary), and shall contain large and well-equipped laboratories for a small army of researchers. Not only is the donor prepared to do this, but the offer includes the provision of the necessary and up-to-date accommodation for the complete Medical School buildings which are not already included in the generous gifts of Sir William James Thomas." This gift, therefore, places the College in the position of having funds available for the whole of the buildings required for a complete Medical School. The conditions attached are that the College provide sites for the School of Preventive Medicine, and for the departments of anatomy, surgery, medicine, and midwifery; that certain specified grants shall be made available for the building and equipment; and, most important of all, that the grant made by the Treasury, in addition to the present grant of £1,500, shall be, in Sir W. Osler's opinion, adequate for a first-rate Medical School. Sir William James Thomas's gift of £30,000 has thus borne splendid fruit, as the voluntary system has again and again shown that such gifts will. The donor, Colonel Bruce-Vaughan states, has been much influenced by the belief that the provision of up-to-date research laboratories would do more than anything else to secure the annual grant of £3,000 to the school, which is available from the penny per insured person in Wales for research purposes. Certainly never have voluntary workers done more to deserve official benediction than the hospital men of Cardiff of late years.

### THE JORDAN LLOYD MEMORIAL AT BIRMINGHAM.

At the Queen's Hospital, Birmingham, a marble bust of the late eminent surgeon, Mr. Jordan Lloyd, has been unveiled, and one of the wards also named after him. The late Professor held the post of surgeon to the hospital for thirty years, and Professor J. T. J. Morrison, senior surgeon to the hospital, paid a very remarkable tribute to his extraordinary powers of diagnosis and operative skill, and all Birmingham owed a debt to his memory, which was especially dear to the three institutions where he worked—Queen's Hospital, the Children's Hospital, and the Infirmary. No surgeon in England, continued the speaker, had performed a larger number of major operations. This bust, which is the work of Mr. A. Toft, the sculptor, has been placed in the entrance hall of Ward I.

### BRADFORD'S LATEST HOSPITAL.

The Health Committee of Bradford appears to be a peculiarly active body, for it has opened a department—really a small hospital—for the treatment of ophthalmia neonatorum and the operative treatment of ear, throat, and nose diseases. The quarters of the new institution are the isolation block of the Fever Hospital in Leeds Road, which has been erected only a few years, and which from its situation is completely separated from its parent hospital. The alterations have resulted in two large wards, with seven beds in each, and one small ward accommodating two mothers and their infants, making a total of sixteen beds. There is also an up-to-date operating theatre, a small dispensary and laboratory, and ward kitchen. The staff consists of the hospital superintendent, Dr. Kitchin, a resident assistant medical officer, Dr. Cherry, a matron, and four nurses and one servant. The Health Committee, at a visit of inspection to their latest creation, received a brief address from the acting chairman, Mr. H. Thornton Pullan, who reminded those present that ophthalmia neonatorum had now been made notifiable by the Local Government Board, and as the Health Committee were advised that the disease was curable they were resolved to try to cure it. The other cases were those requiring operative treatment for which the local authority had made no provision, and only about half of which were being treated elsewhere. A beginning has thus been made to secure adequate treatment for children in Bradford before and below the school age.

### WOMEN SUFFRAGISTS AS A SOURCE OF INCOME.

The suggestions of Dr. Charles Mercier are always ingenious and logical—too logical, indeed, for it is as much the beginning of wisdom as the mark of cowardice which makes humanity suspicious of carrying out logical courses to their bitter end. His latest attempt to provide " a short way with suffragists " is to treat them as outlaws, on the logical ground that " the punishment would fit the crime to a nicety," since those who defy the law would be deprived of its protection. Hospital officers will note with interest and amusement one aspect of the picture which Dr. Mercier proceeds to draw of the effects of outlawing—that, namely, by which debtors of the outlaw might prefer to pay the debt to someone else, as, for instance, he suggests, to a hospital for women. This calls up curious vistas of the sources of income by which hospitals may benefit in the future. But we believe that Dr. Mercier's suggestion has one practical objection, which probably would prove fatal to its success. By making a woman an outlaw you make her a martyr at the same time, and lend all the appeal of helplessness to her condition. Very few, debtors, we fancy, would dare to take advantage of their legal freedom, and public opinion would make every debt to an outlawed woman a debt of honour, far more strongly binding on the debtor than any process of law is to-day; in which case, women's hospitals would not benefit much by the ingenious suggestion. What are our readers' views?

### A TRAGIC MEDICAL SUICIDE.

The word " tragic " can be applied without rhetoric to the suicide of a medical man, whose professional conscientiousness accused him of a mistake of which his colleagues after his death have acquitted him. Yet such a situation was revealed last week at an inquest at West Bromwich on the death of Dr. H. B. W. Plummer, whose body was found in the bath at his house a few hours before an inquiry was to be held on a patient whom he had attended in her confinement. The story is told in a letter which the deceased doctor addressed to the Coroner, from which we take the following: " I cannot face the disgrace I have brought on myself and my profession. The *post-mortem* this afternoon satisfied me that I was directly responsible for poor Mrs. Dyer's death. . . . No one who must have acted as I did has any right to live or be a member of a profession of which I have always hoped to be a credit." Dr. Parsons, assistant physician at Birmingham General Hospital, stated that the inquiry revealed nothing to imply that the patient's death was due to professional rough handling. Dr. Plummer, he added, had done his best, but an unbalanced mind had led him to take a distorted view of his responsibility. A verdict of " Suicide while of unsound mind " was returned.

### SECRETARIAL APPOINTMENTS IN BIRMINGHAM.

It is not always, or even frequently, possible for a retiring hospital secretary and general superintendent to retain an official connection with his institution after his resignation has taken effect.

This, however, has occurred at the Birmingham and Midland Eye Hospital in the case of Mr. Charles A. Mason, who tendered his resignation of the post of general superintendent towards the close of last year. Mr. Mason, who had held this position for eight years, has accepted a seat on the committee, which will therefore still have the benefit of his experience and advice. His successor in the general superintendentship is Mr. J. W. Pearce, who has been promoted after working in connection with the secretary's office for thirteen years as collector. The new collector is Mr. A. G. Milward.

### THE HOSPITAL DEADLOCK IN BLACKPOOL.

We drew attention in our issue of February 14 to the threatened resignation of the honorary medical staff of the Victoria Hospital, Blackpool, through a dispute which arose between the medical officers and the committee over the dismissal of two sisters. That resignation duly took effect, and in the meantime efforts have been made to secure medical men from any quarter, with varying success. A new development, however, has now occurred, and as a result of a meeting of the medical profession in Blackpool the following resolution, which has been signed by forty-two medical men, has been forwarded to the board of management: "We, the undersigned members of the medical profession resident in Blackpool, being persuaded that the attitude of the board of management to the late members of the honorary medical staff is prejudicial to the interests of the patients and of the town generally, pledge ourselves not to accept any appointment on the Victoria Hospital staff until there is a complete reorganisation of the board of management, and feel that all future appointments should be open to the profession generally." The forty-two signatures are believed to represent the whole of the medical profession in Blackpool, except the four doctors now forming the hospital's staff, and two who are absent from home. Mr. Riding, the honorary treasurer of the hospital, however, still declares that the institution will be unaffected by the attitude of the local profession.

### THE MEDICAL STAFF'S CONTENTION.

The doctors, however, are not letting the matter rest, and the Honorary Secretary of the Blackpool Division of the British Medical Association has issued a statement, from which we take the following: "During the discussion respecting the appointment of a house surgeon to the Victoria Hospital, Blackpool, in November last, the hon. secretary of the medical board, Mr. W. B. Richardson, who is also a member of the board of management, expressed the opinion to the hon. secretary of the hospital, Mr. T. Loftos, that before proceeding with the appointment there would have to be changes both in the quality and quantity of the food as at that time supplied to the nursing staff. A communication was passed on to the chairman of the board, who at the next monthly meeting raised the question, with the result that a sub-committee with plenary powers was appointed to investigate matters. At the first meeting of this sub-committee the three sisters and a senior probationer were summoned and requested to answer questions relative to the food supply. At a later meeting of this sub-committee—January 2, 1914—the two senior sisters were separately called out of the operating theatre and told that 'in the best interests of the hospital it was deemed advisable they should send in their resignations; or, if they preferred it, they could have a month's money and leave at once.'" Out of the refusal of the board of management to agree to the medical board's request to reconsider their decision has grown the present dispute. The staff's view, in short, is that the request to the sisters to resign was tantamount to their dismissal for telling the board, when ordered to do so, the truth about the hospital's food supply. The staff further state that the sisters had not volunteered any evidence, though the correctness of their statements might be inferred from the changes made in the dietary. The staff believe that the sisters were made the scapegoats of the person or persons responsible for the unsatisfactory condition of the food. Where are the governors?

### NEW SECRETARY FOR THE NEW HOSPITAL FOR WOMEN.

Another important change in the staff of the New Hospital for Women, Euston Road, has been caused by the retirement of Miss M. M. Bagster from the office of secretary, and the appointment of Miss Imogen H. Murphy in her place. The report presented at the recent annual meeting recalled that Miss Bagster became secretary in 1885 and said: "In addition to her secretarial work, the hospital owes entirely to her initiative the establishment and maintenance of the Cottage Home for Convalescents at Aldbury, which has been much appreciated by a long succession of patients. The committee feel that they cannot express their opinion of Miss Bagster's services more satisfactorily than by putting on record the following resolution, which was unanimously passed after her resignation was accepted: The committee desire to record their very sincere regret that, after loyal service to the hospital for nearly twenty-eight years, Miss Bagster feels that the time has come to resign the post of secretary. They wish to express their appreciation of the efficient manner in which she has always discharged her duties, the deep sense of the devotion she has ever shown to the work that has been carried on at the hospital, and their grateful consciousness of the zeal and single-mindedness that have been the mainsprings of her actions during the whole time of the tenure of this office."

### THE PUNISHMENT OF INFIRMARY BOYS.

Some time ago we published the verdict and damages given to Dr. W. J. C. Keats, medical superintendent of the Camberwell Infirmary, in the first of a series of libel actions he was bringing against certain newspapers and other persons, arising out of various allegations made by the latter with reference to the punishment of certain convalescent boys. Last week in the action which Dr. Keats brought against Mrs. C. A. R. Bracey-Wright, otherwise known as the Countess de Lormet, and her son, Mr. W. H. C. Bracey-Wright, for an alleged libel contained in an election address, the jury stated that there was no prospect of their agreeing upon a verdict, and Mr. Justice Scrutton discharged them, saying that, having regard to the state of the cause list, if the case were entered for trial it would be heard in a very short time.

### MR. RUNCIMAN ON MEDICAL CERTIFICATES.

Replying to a deputation from a friendly society last Saturday at Dewsbury, Mr. Runciman stated that the increased expenditure since the Insurance Act had been passed had brought the total expenditure to millions more than had been estimated. He went on to say that the granting of medical certificates in a loose way had penalised not only the National Scheme but also the Friendly Societies, and that the sooner this loose way was given up the better. The position could not be left as it was, but whether the extension of medical referees or the setting up of a system of State medical service would be the better remedy it was not for him to say. The Government, he added, was watching with grave anxiety this aspect of National Insurance. No doubt alarm of insolvency has put the crowning touch to the Government's anxiety, but it would be unfair to place the whole blame, or more than a small part of it, on the careless granting by medical men of certificates of incapacity. This, no doubt, has some part in it, but it is more a symptom than a cause, the cause being the vast amount of hitherto unsuspected sickness which the Insurance Act, satisfactorily in the long run, has revealed, together with the inadequacy of medical benefit, which, through the absence of prompt institutional and skilled treatment, allows disablements to become, one might almost say, chronic, and vastly lengthens the period during which insured persons remain upon the funds of their approved societies.

### THE SOLDIERS' DAUGHTERS' HOME, HAMPSTEAD.

Readers of our issue of March 7, who will remember our exposure of the sanitary condition of the Soldiers' Daughters' Home, Hampstead, will be interested in the report on its condition which Dr. F. E. Scrase, medical officer of health, presented to the Hampstead Borough Council last week. After describing the cases of infectious disease which have been occurring till lately in the institution since November 20 last, and the unsatisfactory reports of the sanitary inspectors, who made special visits, and finally the bacteriologist's report, Dr. Scrase went on to say that the committee decided to empty the Home and to secure a building elsewhere to which the inmates could be transferred pending its renovation, which has been agreed upon. The medical officer expressed the view that the continued presence of diphtheria in the Home was due to carrier cases, though the number of sore throats pointed to there being "something connected with the building that has caused the inmates to become susceptible to diphtheria, and has made it a suitable ground on which to flourish once the disease had been introduced." The architect of the Home has estimated that the requirements of the medical officer of health would cost £4,000 to carry out. When the above report came before the Hampstead Borough Council, Councillor A. B. Weaver stated that all the inmates would be transferred to a Jewish Home at Streatham. But the matter cannot be left here.

### THE MISTAKE OF TINKERING.

Radical as are the innovations demanded by the medical officer of health, we believe that it would be a thoroughly mistaken policy to tinker with the existing Home. Rebuilding in the long run has almost always proved less expensive than reconstruction, and the present authorities have not shown sufficient administrative ability to justify public confidence in undertaking such a work. The proper course would be to pull down the existing building, sell the site, and either rebuild or find suitable quarters elsewhere. Such a policy, we believe, would reassure the subscribers that the conditions of the past had been permanently put an end to.

### THE NELSON HOSPITAL'S NEED FOR EXTENSION.

The year 1913 has passed very satisfactorily for this institution, for while the accounts presented showed for the first time (since 1912 was a year of transition) that the increased expenditure was being met, all fears that extreme difficulty would be found in raising an income sufficient for the enlarged building appear groundless. Comparing 1911 with 1913, the beds have increased from fourteen to twenty-six; the total expenditure from £673 in 1911 to £1,378 in 1913. The corresponding income has risen from £685 to £1,415. The new hospital was intended for twenty-four beds and cots; but on several occasions during 1913 twenty-eight patients were accommodated, and on the evening of the meeting last week there were actually twenty-nine patients in hospital. All this points to the probable early need of the extension, a gratifying proof that the rebuilding is justifying itself and that the new hospital is filling a growing need in the district.

### COVENTRY HOSPITAL AND TYPHOID CASES.

The admission of typhoid cases to the Coventry and Warwickshire Hospital again came up for discussion last week on the recommendation of the house committee that a limited number be received. Three years ago, in February 1911, it was decided not to admit typhoid cases until further accommodation was available; but, in supporting their re-admission, Mr. W. J. Wormell said that provision had now been made, and that the medical staff unanimously favoured the proposal. Mr. E. M. Iliffe explained in answer to a question that the object of the committee was to give experience of typhoid cases to the nurses, who without it were not considered fully trained. The house committee's recommendation was carried. At the same meeting a graceful and deserved acknowledgment was accorded to a Hospital Saturday worker, Mr. Henry Delo, who has been a member of the Hospital Saturday Committee for thirty-seven years and has represented one firm for the whole of that period. On Mr. T. H. S. Nichols' suggestion, advantage was taken of the law by which anyone who had served the hospital well could be appointed a life governor, to pay this compliment to Mr. Delo as a unanimous official recognition of his long period of work.

### THE JESSOP HOSPITAL AND MATERNITY BENEFIT.

THE experience of the Jessop Hospital, Sheffield, under the Insurance Act is worth putting on record, for it is one which was not anticipated. A considerable falling off in the number of patients treated is recorded in both the in- and out-patient departments, and the explanation put forward is that the 30s. maternity benefit has enabled many patients to remain at home who would formerly have come into the hospital. The effect on the hospital of this decrease is anxiety lest, if the decrease were maintained, it would affect the hospital's reputation as a training school for certificated midwives by limiting the amount of experience obtainable. But were the above explanation wholly sufficient to account for the decrease other women's hospitals would be similarly placed, and, therefore, the different institutions would be on the same level, though with less to offer than before. It seems, however, that a wholly erroneous impression has gained wide currency in Sheffield that by coming to the hospital maternity benefit would be forfeited; and the board of management have had some difficulty in making it as widely known as was necessary that no part of the 30s. is taken by the hospital, the patient or those of her family entitled to it receiving the benefit in full. It would be interesting to know how far the Jessop Hospital's experience has been shared by other women's hospitals, and we should be glad to receive and publish other information on this point.

### THE INSURANCE ACT AND WOMEN DOCTORS.

Is the Insurance Act proving beneficial to women doctors in search of resident medical posts? This question is suggested by the unanimous decision of the Dewsbury Infirmary board to suggest to the medical staff the advisability of appointing a woman doctor as resident house surgeon. This post has been vacant now for some time, and it is stated that the board have experienced a difficulty in filling the resident posts ever since the passing of the Insurance Act. The one or two applicants that have appeared have subsequently withdrawn, with the exception of women doctors, who, it was pointed out by Mr. R. Armstrong, had many reasons in favour of their appointment, the number of women and children in the female wards being among them. The matter has, therefore, been referred to the honorary medical staff. Probably though many women doctors have individually a large following, they have rarely if ever commanded sufficiently widespread confidence to gain much out of contract practice.

### AN INDIAN HOSPITAL ADMINISTRATOR.

THE late Sir Arthur Branfoot, K.C.I.E., whose death occurred last week, had a wide experience of Indian hospitals. A "Guy's" man, who entered the Indian Medical Service in 1872, he became resident surgeon at the General Hospital, Madras, and Professor of Hygiene at the Madras Medical College. His interest not merely in midwifery, but in the institutional conditions of women's treatment was here developed, and taken advantage of in his appointment as superintendent of the Government Maternity Hospital. In 1898 he became principal medical officer of the Rangoon District of Burma, to return to Madras in 1901 as Surgeon-General to the Government. He retired in 1903, and became President of the Medical Board at the India Office, and a member of the Advisory Board for Army Medical Services. This brief summary of a long and useful career indicates how wide is the scope offered to active brains and energies by the profession of hospital administrator if the ideal of personal service be held through life.

### PERCHED AROUND A BILLIARD TABLE.

THE Southwark Board of Guardians have decided to increase the salaries of the medical staff at the infirmary. That of the assistant medical superintendent is to be increased from £180 a year to £200, that of the second assistant medical officer from £140 to £180, and that of the third from £120 to £180, the same as the second assistant medical officer. Considerable difficulty has been experienced in obtaining candidates for the positions, and it has been found that they very rarely stay more than a year. Six months ago the Guardians gave the medical staff permission to place a convertible billiard table in the committee room at the infirmary, and at the last meeting the staff asked the Board to allow it to remain a further period of six months. It was stated that a permanent and not a convertible table had been placed in the room, and that when the committee met a board was put on the top. This raised the temporary table a few inches, which has caused the Guardians a little inconvenience. They objected to being perched up like dolls, as one member described them, although it was pointed out by Mr. J. Osborn, the vice-chairman, that for the amusement of the staff during the long hours they were forced to be at the infirmary the Guardians should forego their convenience. His appeal did not influence his colleagues to continue the concession granted to the staff, for by eleven votes to eight they refused permission for the billiard table to remain. In view of the difficulty they have experienced in filling up the medical posts their action may yet be regretted by themselves.

### A VOICE FOR HOSPITAL PHARMACISTS.

Mr. Herbert Skinner, the well-known pharmacist to the Great Northern Central Hospital, London, has been nominated as a candidate for the Council of the Pharmaceutical Society of Great Britain. The list of nominations was closed on the 18th inst., and a very keen contest was anticipated. Mr. Skinner expected to have the official support of the Public Pharmacists' and Dispensers' Association, of which organisation he is a member, and, in addition, of the North London Pharmacists' Association, as he is the secretary to that body. Up to the present, institutional pharmacists have had no direct representative to voice their opinions at the Council of the Pharmaceutical Society.

### THE SALARIES OF DISPENSERS.

Difficulty is being experienced in obtaining the services of dispensers for hospitals and other institutions at the salaries which were ruling a year or so ago. There does not appear to have been an all-round demand for much higher salaries by dispensers already employed, but the difficulty arises when it becomes necessary to fill a vacancy, and it seems probable that before long institutions will find it almost imperative to raise the rate of pay of these officers. This is, of course, due to the operation of the Insurance Act, which caused an enormous increase in the amount of dispensing done by chemists, and consequently a demand by chemists for competent assistants; the salaries paid by chemists are, generally speaking, above those paid by institutions, and hence the difficulty of obtaining dispensers at the old rates.

### A SUCCESSFUL SUBSIDIARY ORGANISATION.

One of the most valuable results of the League of Mercy has been not merely the gospel of personal service which it has preached and the money which it has collected, vastly important as both are, but the inspiration of many hospital movements which directly or indirectly owe their origin to its example. Of the many in and out of London which might be mentioned, the Bedfordshire Hospital Guild is one worth attention by all hospital committees and secretaries who wish to see how their hospital's income may be increased by local organisation. The objects of the Guild, which is now seven years old, are to increase the hospital's income by a number of small subscriptions, and also to add to its utility by doing supplementary charitable work. It arranges the house collections during hospital week, organises sales and entertainments throughout the county, makes garments and supplies linen for the hospital's use, and takes part in after-care work by collecting and managing a fund for supplying surgical instruments and sending patients to convalescent homes. The latest report shows that £646 was collected by the Guild through house-to-house collections in the town and county. The district is divided into twenty divisions, but for some reason or other only thirteen contributed. This means, we take it, that the divisions are made on the map, but that all of them are not in the hands of capable organisers. But we have said sufficient to show its value, both as a source of income and, what is equally important, as a centre of activity, which hospitals wanting a working model of a comprehensive subsidiary organisation would do well to study.

### THE OPENING OF THE PANAMA CANAL.

It was a happy thought of Sir William Osler's to suggest that the medical profession should give a complimentary dinner to Surgeon-General Gorgas, sanitary officer of the Panama Canal Commission, on his arrival in England for a brief visit last week. The dinner, which took place on Monday night, was presided over by Sir Thomas Barlow, P.R.C.P., and speeches were made by Lord Bryce and the American Ambassador. Lord Bryce, in an amusing reference to the mission of Mr. Wycliffe Rose to Egypt, the Straits Settlements, and Ceylon, as representing the International Health Commission of America, remarked that medicine was the only profession, so far as he knew, which laboured incessantly to destroy the reason for its own existence. In reply to the toast of his health, which was proposed by Sir Havelock Charles, Surgeon-General Gorgas stated that the Panama Canal would be formally opened in January. Notwithstanding the prosperous schools of tropical medicine in this country, the hospital world needs to be reminded from time to time of its relation to medicine in other countries and hemispheres, and this dinner, apart from its personal tribute to Surgeon-General Gorgas, had a value also in this respect.

### THIS WEEK'S DRUG MARKET.

Business has been rather quiet during the week, and there have not been many price changes of particular note. The pronounced scarcity of the supplies of citric acid has led to an advance in price not only of the acid itself, but also of the citrates—potassium citrate, sodium citrate, etc. The present price of citric acid is fully 30 per cent. above what used to be considered a fair quotation and twice the lowest price ever reached; this product is in large demand at the present season, as it is used in considerable quantities in the manufacture of effervescent saline preparations, but owing to the scarcity and dearness of the acid it is probable that tartaric acid, which is something like half the price, is used in its place in the manufacture of the cheaper preparations. There has been little change in the position of cod-liver oil; the fishing has continued to yield good results, but there has been no further decline in price and a fair business has been done. Essence of lemon has again tended downwards in price. The price of castor oil is rather lower. The value of opium is well maintained, and morphine is unchanged.

### TO OUR READERS.

Contributions are specially invited from any of our readers to these columns. They should deal with topical subjects and news. They must be authenticated for the information of the Editor only. The minimum payment if published is 5s. There is no hard-and-fast rule as to space, but notes of about twenty lines in length are preferred.

